# Molecular mapping of the grain iron and zinc concentration, protein content and thousand kernel weight in wheat (*Triticum aestivum* L.)

**DOI:** 10.1371/journal.pone.0174972

**Published:** 2017-04-06

**Authors:** Gopalareddy Krishnappa, Anju Mahendru Singh, Swati Chaudhary, Arvind Kumar Ahlawat, Santosh Kumar Singh, Ram Bihari Shukla, Jai Prakash Jaiswal, Gyanendra Pratap Singh, Ishwar Singh Solanki

**Affiliations:** 1 Division of Genetics, ICAR-Indian Agricultural Research Institute, New Delhi, India; 2 Division of Crop Improvement, ICAR- Indian Institute of Wheat & Barley Research, Karnal, Haryana, India; 3 Govind Ballabh Pant University of Agriculture and Technology, Pantnagar, Uttarakhand, India; 4 ICAR- Indian Agricultural Research Institute, Regional Station, Samastipur, Bihar, India; Institute of Genetics and Developmental Biology Chinese Academy of Sciences, CHINA

## Abstract

Genomic regions responsible for accumulation of grain iron concentration (Fe), grain zinc concentration (Zn), grain protein content (PC) and thousand kernel weight (TKW) were investigated in 286 recombinant inbred lines (RILs) derived from a cross between an old Indian wheat variety WH542 and a synthetic derivative (*Triticum dicoccon* PI94624/*Aegilops squarrosa* [409]//BCN). RILs were grown in six environments and evaluated for Fe, Zn, PC, and TKW. The population showed the continuous distribution for all the four traits, that for pooled Fe and PC was near normal, whereas, for pooled Zn, RILs exhibited positively skewed distribution. A genetic map spanning 2155.3cM was constructed using microsatellite markers covering the 21 chromosomes and used for QTL analysis. 16 quantitative trait loci (QTL) were identified in this study. Four QTLs (*QGFe*.*iari-2A*, *QGFe*.*iari-5A*, *QGFe*.*iari-7A* and *QGFe*.*iari-7B*) for Fe, five QTLs (*QGZn*.*iari-2A*, *QGZn*.*iari-4A*, *QGZn*.*iari-5A*, *QGZn*.*iari-7A* and *QGZn*.*iari-7B*) for Zn, two QTLs (*QGpc*.*iari-2A* and *QGpc*.*iari-3A*) for PC, and five QTLs (*QTkw*.*iari-1A*, *QTkw*.*iari-2A*, *QTkw*.*iari-2B*, *QTkw*.*iari-5B* and *QTkw*.*iari-7A*) for TKW were identified. The QTLs together explained 20.0%, 32.0%, 24.1% and 32.3% phenotypic variation, respectively, for Fe, Zn, PC and TKW. *QGpc*.*iari-2A* was consistently expressed in all the six environments, whereas, *QGFe*.*iari-7B* and *QGZn*.*iari-2A* were identified in two environments each apart from pooled mean. *QTkw*.*iari-2A* and *QTkw*.*iari-7A*, respectively, were identified in four and three environments apart from pooled mean. A common region in the interval of *Xgwm359-Xwmc407* on chromosome 2A was associated with Fe, Zn, and PC. One more QTL for TKW was identified on chromosome 2A but in a different chromosomal region (*Xgwm382-Xgwm359*). Two more regions on 5A (*Xgwm126-Xgwm595*) and 7A (*Xbarc49-Xwmc525*) were found to be associated with both Fe and Zn. A QTL for TKW was identified (*Xwmc525-Xbarc222*) in a different chromosomal region on the same chromosome (7A). This reflects at least a partly common genetic basis for the four traits. It is concluded that fine mapping of the regions of the three chromosomes of A genome involved in determining the accumulation of Fe, Zn, PC, and TKW in this mapping population may be rewarding.

## Introduction

Both plants and human beings require an optimum daily intake of protein, vitamins, and micronutrients for their normal physiological and biochemical activity. The leading risk factor for health loss, of course, is undernutrition, particularly for those who live in low and middle-income countries [1]. Moreover, a majority of foods fail to deliver consistently the required quantity of micronutrients for human body [2]. Approximately one-fourth of the population across the globe suffers from anemia caused by Fe deficiency [3], which resulted in the loss of around 46,000 disability adjusted life years in 2010 [4]. Insufficient Fe intake affects physical growth and cognitive ability [5], so also the reproductive ability and work efficiency [6]. A sizeable population (around 17.3%) across the globe is also facing Zn deficiency related diseases [7]. Insufficient intake of Zn causes excessive weight loss, depression, psychosis, diarrhea, impaired growth and development, altered reproductive biology, gastro-intestinal problems and impaired immunity [8]. Grain protein is an important trait that determines the nutritional quality and baking properties of wheat. Protein is an essential component of cells and it supports muscle growth, immune and enzymatic reactions; protein-energy malnutrition causes marasmus and kwashiorkor. Protein and micronutrient malnutrition continue to be a major health burden in developing countries, where pregnant women and young children are particularly vulnerable [9]. Grain yield is a complex trait and is influenced by a number of component traits including spike number per square meter, TKW, and number of grains per spike. High TKW can improve the flour yield and affect the milling quality of wheat grains [10].

Protein and micronutrient deficiency can be overcome by consuming a diverse diet, but it can not be afforded by a majority, particularly in developing and underdeveloped countries, where malnutrition is a major problem. The other alternatives are supplementation and fortification but these are difficult to sustain on a long-term basis. These approaches also have other shortcomings such as non-availability of the fortified food to the needy people, so also the lack of affordability, particularly for rural poor [11]. Plant breeding to improve the nutritional value of crop plants also termed as ‘biofortification’, has been recognized as an economical and sustainable strategy that can be useful as a complementary solution to the problem of malnutrition. Biofortification has been recognized among the five topmost solutions to the problem of micronutrient malnutrition in the Copenhagen consensus, 2008 [12]. Currently, development of nutrient dense staple food crops is one of the prime research areas for the scientific community. High Fe beans, high Zn rice, Pro-vitamin A rich cassava, high Fe pearl millet etc. are some examples that speak of the feasibility and success of this strategy [13].

Wheat alone constitutes nearly one-third of the total cereals consumed in the world [14] and is a staple crop of many nations including India. Thus, wheat is an important source that can meet a major proportion of the required nutrients. However, major wheat based diets fail to deliver the required quantity of protein and minerals to humans. Therefore, it is imperative that wheat varieties with improved contents of Fe, Zn and PC be developed to alleviate hidden hunger among people who consume mainly cereals.

Grain accumulation of micronutrients is a physiologically complex trait [15]. We still lack a clear understanding of the processes which control Fe and Zn translocation in the plant system and its accumulation in the grain. While this understanding will be very useful in speeding up the breeding process, identifying loci involved in grain accumulation trait *per se* can also be an effective approach. For this, an extensive exploration of plant genetic resources for identifying those with higher mineral accumulation in seeds is indispensable; critical also is the understanding of the effect of environment on traits expression and so also its interaction with genotype [16–17]. This would aid in identifying loci or genomic regions (QTLs) harbouring genes for the accumulation of high Fe and Zn levels in the wheat grain and would allow breeders more efficiently to develop biofortified cultivars by using closely linked molecular markers to screen and select the most favorable genotypes. This indirect marker-aided selection (MAS) is more important for difficult to breed traits like grain Fe and Zn and PC, which exhibit high genotype-environment interaction [18–24].

Recently, QTLs were identified in different mapping populations of wheat. A major QTL, *Gpc-B1* from wild emmer wheat (*Triticum turgidum* ssp. *dicoccoides*) was mapped on chromosome arm 6BS [25]. It was cloned and said to be effective in improving Fe, Zn, and PC by 18%, 12%, and 38%, respectively [26–27].

The type and the size of a mapping population and the frequency and distribution of molecular markers on framework linkage map are considered to be important determinants for identification of a QTL. However, in most studies on identification of QTLs for Fe [28–35], Zn [28–30, 32–37], PC [28, 32–33, 38–39], and TKW [40–45] the size of mapping population was limited. The present study was aimed to identify the genomic region(s) associated with grain Fe, Zn, PC, and TKW in a set of the 286 RILs.

## Materials and methods

### Genotypes and environments

Materials consisted of 286 RILs developed by single seed descent method from a biparental cross between an Indian bread wheat cultivar WH542 and a synthetic derivative (*Triticum dicoccon* PI94624/*Aegilops sqarrosa* [409]//BCN) from CIMMYT, Mexico. The RILs were developed from F_2_ by single seed descent method and maintained at Grain Quality Laboratory, ICAR-IARI, New Delhi, India. The RILs (in F_7_) were evaluated at three different locations, two belonging to the North Western Plains Zone (NWPZ) and one to the North Eastern Plains Zone (NEPZ) during the winter (rabi) season of the years 2012–13 and 2013–14. The locations in NWPZ include Delhi (ICAR-Indian Agricultural Research Institute, Research Farm, New Delhi, 28° 38’N, 77° 9’E, 228.6 m AMSL) and Pantnagar (Govind Ballabh Pant University of Agriculture and Technology, Research Farm, Uttarakhand, 29°N, 79°31’E, 243.8 m AMSL). The third location, Pusa Bihar at ICAR-IARI, Regional Station, Research Farm, Samastipur, 25° 14’N, 87° 2’E, 62.5 m AMSL belonged to the NEPZ. The crop was timely sown under irrigated conditions in first fortnight of November at all the locations in both the years. The genotypes were planted in a randomized complete block design (RCBD) with two replications per entry and two rows (5 m length) per replication with a plant-to-plant spacing of 10 cm and row-to-row spacing of 25 cm. Standard agronomic practices were followed for raising the crop.

### Micronutrient analysis

The mapping population consisted of 286 RILs and two parents. The entire set was grown in two field replications. After harvest of 25–30 spikes from each replicate, the spikes of a replicate were bulk threshed in a clean cloth bag by beating with a wooden stick and the grain was separated from husk in a plastic *chaaj*. Care was taken at every step to avoid dust and metal contamination and sub sampling was done; finally three samples were drawn. Grain iron and zinc in each of these samples was estimated and the averaged value was considered the value of one replication. The grain samples for Fe and Zn were measured using Energy Dispersive X-ray Fluorescence (ED-XRF). The XRF machine model X-Supreme 8000 (M/s Oxford Inc, USA) was used for the purpose. This machine was calibrated using the glass beads based values. The glass beads contain standards with different concentrations of different minerals (which are earlier measured with ICP-MS by Dr. James Stangoulis; Technical coordinator, HarvestPlus at Flinders University, Australia). The glass beads were provided under the HarvestPlus Programme to AMS associated as a NARS partner. The same method has been used by others recently [21, 28, 30]. Thus, 3456 data points were generated for iron and zinc.

### PC and TKW

The PC in the grain samples as determined by the Kjeldahl method using the Autokjeltech system 3100 from Foss, Tecator, USA. 20 gm grain sample was ground using cyclotech mill (Foss Tecator, Sweden). 10 ml concentrated H_2_SO_4_ and 4.5 g of catalyst mixture was added to the 0.5 g of grain sample. The samples were digested at 450°C for 30–45 minutes till contents were clear. After cooling, 70 ml distilled water was added to the digestion tubes, the digested contents were distilled with the distillation system. The PC was obtained by multiplying with a factor of 5.7 and the values were expressed at 12% moisture basis. The moisture content of each of the samples was also simultaneously obtained from Single Kernel Characterization System (Perten, Australia). Each entry was evaluated twice. To record TKW, reading was set at 1000 grains in the Numigral grain Counter and the weight of the grains was recorded with an electronic balance.

### DNA extraction and genotyping

RILs and parental genomic DNA was extracted from the leaves of 21 days old seedlings by following CTAB method of Murray and Thompson [46]. PCR was carried out in a total volume of 15μl, the ingredients include double distilled water(ddH_2_O), 10X buffer (100mM Tris-HCl with pH 8.8; 500mM KCl; 1% Triton X-100; 16mM MgCl_2_), dNTPs, primer, Taq polymerase (Bangalore Genei, India) and DNA. The proportion of ingredients for 15μl PCR mixture for *Xbarc* primer series: ddH_2_O(10.2μl), 10X buffer(1.0μl), DNA(2.0μl), dNTPs(0.5μl), forward and reverse primer(1.0μl) each and Taq polymerase(0.3μl); for *Xwmc* primer series: ddH_2_O(8.4μl), 10X buffer(1.5μl), DNA(2.0 μl), dNTPs(0.6μl), forward and reverse primer(1.0μl) each and Taq polymerase(0.3μl); for *Xcfa*, *Xcfb*, *Xcfd* and *Xgdm* primer series: ddH_2_O(9.1μl), 10X buffer(1.5 μl), DNA(2.0μl), dNTPs(0.6μl), forward and reverse primer(1.0μl) each and Taq polymerase(0.25μl); for *Xgwm* primer series: ddH_2_O(10.2μl), 10X buffer(1.0μl), DNA(2.0μl), dNTPs(0.5μl), forward and reverse primer(0.5μl) each and Taq polymerase (0.3μl). The amplified PCR products were resolved in 3.5% agarose at 120V for 3 hours in TBE buffer. Wherever the resolution was low, the amplified PCR products were separated by electrophoresis on 4% metaphor agarose. PCR amplified products were visualized and photographed on a UV transilluminator (Syngene) for further scoring.

#### Linkage map construction

A total of 714 SSR markers were used for parental polymorphism survey [47–48]. These were chosen such as to cover all chromosome arms of bread wheat genome. The bands generated by SSR primers were scored by giving code ‘A’ for those RIL populations showing the same band as that of WH542 and ‘B’ for those showing the same band size as of synthetic derivative in the case of polymorphic SSRs. Allelic segregation at each of the marker loci was analyzed for segregation distortion from the expected 1:1 ratio in the RIL population using the goodness of fit (χ^2^) test. The markers which exhibited 1:1 segregation pattern were only further used for linkage map construction. Linkage analysis was performed using MapMaker/EXP 3.0 [49–50]. Markers were first grouped using a cut-off recombination value of 0.39 and threshold LOD score of 3.0. Then, the Order command was used to determine a linear order. The Try and Ripple commands were used to add markers to the map and re-test the final marker order. The Kosambi mapping function was used to convert recombination frequencies into map distances.

### Data analysis and QTL mapping

Pearson correlation coefficient was calculated for all the six environments to study the association of Fe, Zn, PC, and TKW in the grains of RILs population. Student’s t-test was used to test the significance of the correlation coefficient. The QTL analysis was performed using adjusted means of the phenotypic trait value and genotyping data using QTL cartographer 2.1 software employing the full-QTL model. The adjusted mean trait phenotypic values were used as input data to detect QTLs. The composite interval mapping method [51–52] was used for identification of QTLs. The test window size was set to 5cM with a walk speed of 2cM and a cut-off probability of 0.05 for deciding the significance of the QTL. LOD threshold for declaring a QTL significant was determined by permutation tests using 1,000 reiterations.

## Results

### Phenotypic variation and association studies

**Fig 1:**
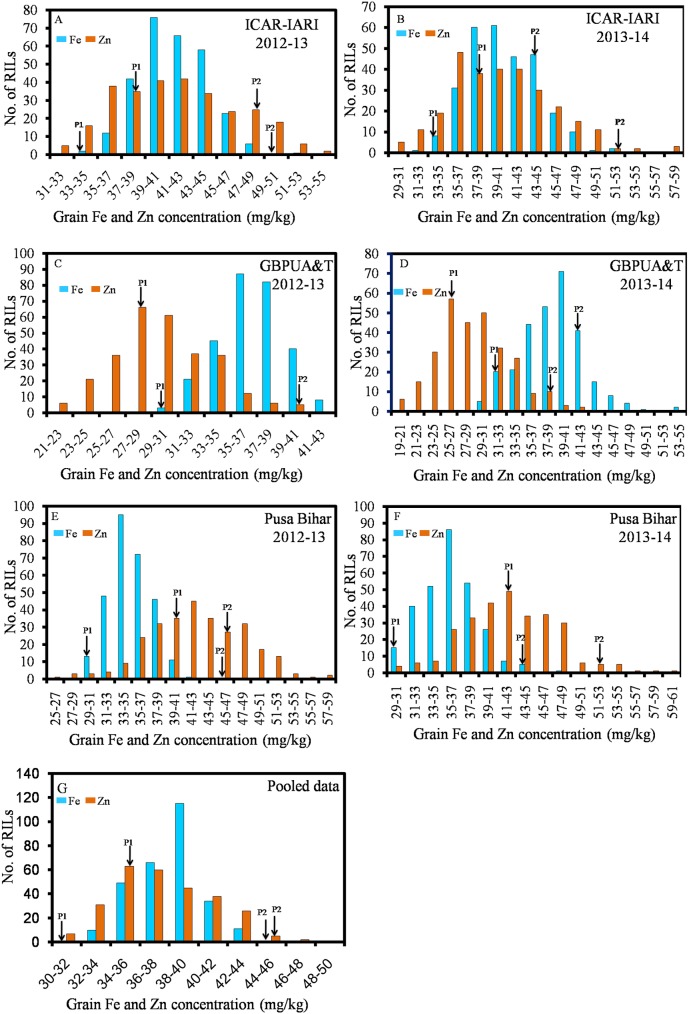
Frequency distributions of grain iron and zinc concentration measured during 2012–13 and 2013–14 in the parents and their RIL population.

**Fig 2:**
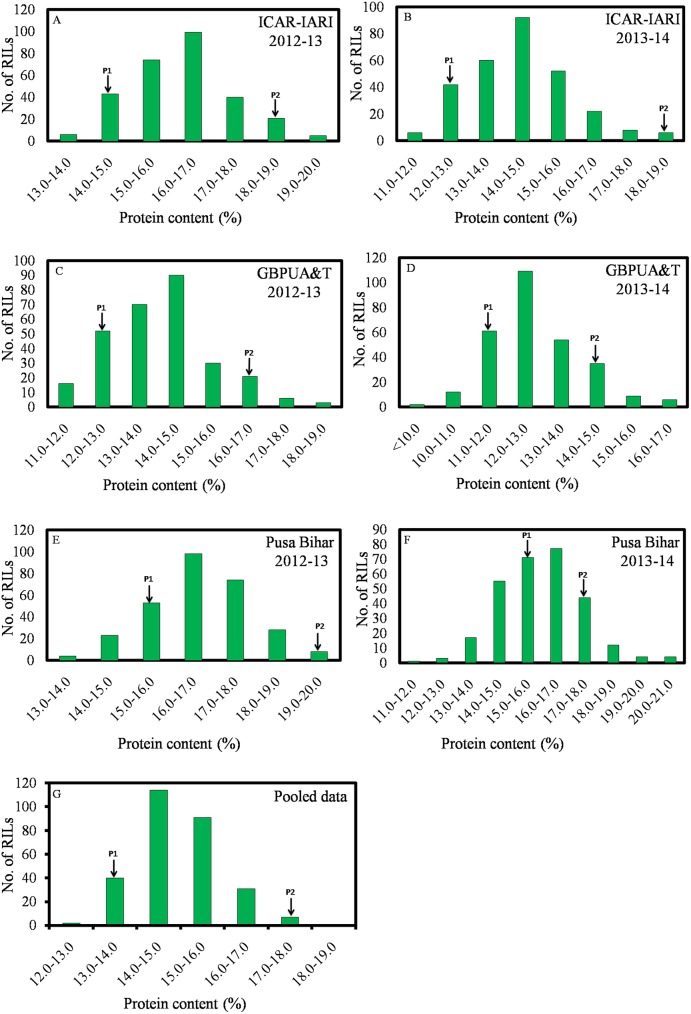
Frequency distributions of grain protein content measured during 2012–13 and 2013–14 in the parents and their RIL population.

**Fig 3:**
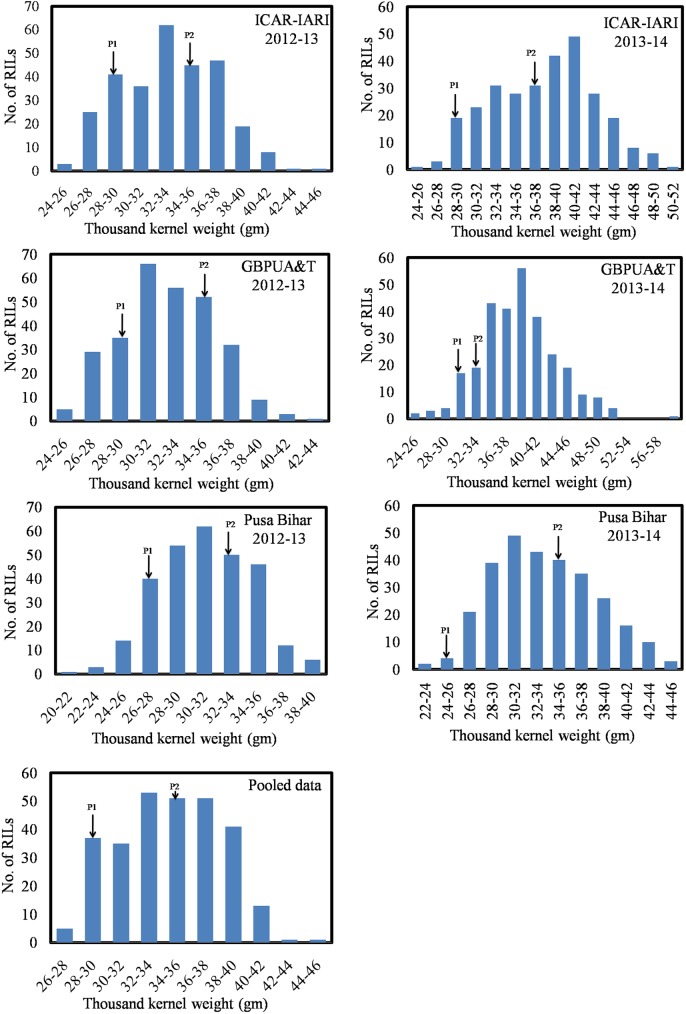
Frequency distributions of thousand kernel weight measured during 2012–13 and 2013–14 in the parents and their RIL population.

The Mean values of Fe, Zn, PC, and TKW are presented in Table 1. Individual mean values of RILs for Fe, Zn, PC, and TKW tested in six environments are given in supplementary tables as S1 Table, S2 Table, S3 Table and S4 Table, respectively. The parents were contrasting for all the four traits. Fe, Zn, PC and TKW, averaged over the replications, years and locations were 31.68mg/kg, 35.07mg/kg, 13.77%, and 28.76gm respectively, for WH542 (henceforth called the ‘low parent’or P1), whereas, the averages of Fe, Zn, PC, and TKW were 45.37mg/kg, 44.39mg/kg, 17.46%, and 34.47gm, respectively, for the synthetic derivative (henceforth called the ‘high parent’ or P2). A wide range of variation was observed among the 286 RILs. The population showed the continuous distribution for Fe, Zn, PC and TKW (Figs 1–3), that for pooled Fe and PC was near normal, whereas, for pooled Zn, RILs exhibited positively skewed distribution. Transgressive segregants appeared for all the four traits and which surpasses the parents and check variety (HD2967). Pearson’s correlation coefficient (r^2^) of Fe, Zn, PC and TKW in the RIL population was determined and are presented in Table 2. The associations were highly significant and positive in most of the environments.

**Table 1:** Mean and range of grain iron, zinc, protein and thousand kernel weight in RIL population grown in rabi 2012–13 and 2013–14 at ICAR-IARI, GBPUA&T, and Pusa Bihar.

Trait/ Environment	WH542(P1)	Synthetic derivative(P2)	HD2967 (Check)	Mean of RILs	Range of RILs
**Grain iron concentration**					
ICAR-IARI 2012–13	33.76	49.60	37.6	41.49±0.17	33.30–51.05
ICAR-IARI 2013–14	33.82	45.00	31.10	40.74±0.21	31.40–51.55
GBPUA&T 2012–13	30.30	45.75	40.02	36.63±0.15	30.00–42.65
GBPUA&T 2013–14	32.11	42.02	41.36	38.68±0.23	27.85–54.60
Pusa Bihar 2012–13	30.00	45.15	35.70	34.92±0.14	29.45–41.85
Pusa Bihar 2013–14	30.04	44.70	34.20	35.86±0.18	29.05–47.75
Pooled mean	31.68	45.37	36.67	38.05±0.13	32.56–44.18
**Grain zinc concentration**					
ICAR-IARI 2012–13	37.68	48.75	36.61	41.63±0.29	30.65–54.50
ICAR-IARI 2013–14	37.22	52.28	34.33	40.56±0.31	29.65–58.85
GBPUA&T 2012–13	27.74	39.32	33.24	29.85±0.22	21.15–41.30
GBPUA&T 2013–14	26.86	38.45	26.43	28.90±0.26	19.30–42.40
Pusa Bihar 2012–13	39.80	45.93	38.27	42.61±0.33	26.45–59.55
Pusa Bihar 2013–14	41.12	51.03	37.40	42.30±0.31	29.75–59.50
Pooled mean	35.07	45.96	34.38	37.64±0.19	30.31–48.36
**Grain protein content**					
ICAR-IARI 2012–13	14.6	18.59	13.70	16.22±0.07	13.75–20.22
ICAR-IARI 2013–14	12.43	18.49	12.04	14.41±0.08	11.45–18.49
GBPUA&T 2012–13	12.80	16.26	13.50	14.07±0.08	11.13–18.63
GBPUA&T 2013–14	11.30	14.60	11.85	12.77±0.07	9.61–16.81
Pusa Bihar 2012–13	15.85	19.06	13.47	16.62±0.07	13.11–19.85
Pusa Bihar 2013–14	15.66	17.75	13.28	15.93±0.09	11.34–21.47
Pooled mean	13.77	17.46	12.97	15.00±0.06	12.78–18.39
**Thousand Kernel weight**					
ICAR-IARI 2012–13	28.14	35.01	36.80	33.23±0.22	24.87–44.42
ICAR-IARI 2013–14	29.09	36.93	38.20	37.97±0.31	24.61–50.63
GBPUA&T 2012–13	29.59	35.08	38.00	32.45±0.20	24.44–42.46
GBPUA&T 2013–14	31.20	33.80	36.50	38.65±0.30	25.42–58.80
Pusa Bihar 2012–13	26.83	32.65	36.20	31.07±0.20	21.82–39.70
Pusa Bihar 2013–14	25.10	35.93	37.39	33.79±0.27	23.34–45.77
Pooled mean	28.33	34.90	37.18	34.53±0.21	27.06–43.05

**Table 2:** Estimates of phenotypic correlation coefficients for grain iron, zinc, protein and thousand kernel weight in RILs of WH542 × Synthetic derivative in rabi 2012–2013 and 2013–14 grown at ICAR-IARI, GBPUA&T, and Pusa Bihar.

	1	2	3	4	5	6	7	8	9	10	11	12	13	14	15	16	17	18	19	20	21	22	23	24	25	26	27	28
1	1	.51**	.43**	.41**	.52**	.51**	.77**	.55**	.41**	.42**	.26**	.36**	.33**	.57**	.55**	.56**	.35**	.34**	.35**	.45**	.59**	.42**	.39**	.40**	.41**	.42**	.41**	.49**
2		1	.42**	.33**	.46**	.47**	.75**	.36**	.55**	.41**	.14*	.21**	.33**	.49**	.45**	.53**	.33**	.31**	.31**	.36**	.51**	.40**	.42**	.44**	.41**	.49**	.42**	.51**
3			1	.36**	.42**	.42**	.68**	.25**	.31**	.45**	.23**	.21**	.22**	.40**	.44**	.43**	.32**	.29**	.30**	.32**	.47**	.26**	.24**	.38**	.30**	.28**	.29**	.34**
4				1	.35**	.37**	.69**	.25**	.28**	.32*	.42**	.21**	.16**	.39**	.38**	.35**	.24**	.30**	.22**	.29**	.40**	.32**	.24**	.29**	.36**	.30**	.24**	.35**
5					1	.49**	.71**	.42**	.40**	.38**	.25**	.37**	.32**	.53**	.57**	.56**	.35**	.32**	.38**	.52**	.61**	.34**	.34**	.39**	.33**	.35**	.36**	.42**
6						1	.75**	.32**	.32**	.40**	.16**	.25**	.52**	.49**	.40**	.53**	.31**	.312*	.26**	.50**	.53**	.42**	.41**	.45**	.42**	.44**	.45**	.52**
7							1	.49**	.52**	.54**	.34**	.36**	.43**	.65**	.63**	.67**	.43**	.43**	.41**	.55**	.70**	.50**	.47**	.54**	.52**	.53**	.50**	.61**
8								1	.44**	.42**	.32**	.37**	.36**	.72**	.53**	.44**	.34**	.27**	.33**	.43**	.53**	.35**	.35**	.33**	.40**	.30**	.28**	.40**
9									1	.43**	.21**	.32**	.44**	.71**	.40**	.43**	.32**	.14*	.31**	.30**	.43**	.38**	.31**	.40**	.28**	.36**	.32**	.40**
10										1	.25**	.31**	.38**	.65**	.36**	.37**	.21**	.23**	.19**	.28**	.37**	.49**	.41**	.40**	.38**	.43**	.38**	.49**
11											1	.23**	.21**	.52**	.26**	.22**	.26**	0.11	0.1	.21**	.26**	.29**	.28**	.14*	.27**	.24**	.21**	.29**
12												1	.46**	.70**	.33**	.23**	.28**	.13*	.29**	.29**	.35**	.29**	.26**	.18**	.21**	.16**	.21**	.27**
13													1	.73**	.30**	.36**	.25**	.19**	.20**	.36**	.38**	.35**	.35**	.29**	.28**	.32**	.29**	.37**
14														1	.54**	.50**	.42**	.26**	.36**	.47**	.57**	.52**	.48**	.42**	.44**	.43**	.41**	.54**
15															1	.63**	.53**	.40**	.58**	.58**	.83**	.14*	.20**	.22**	.29**	.25**	.24**	.27**
16																1	.44**	.43**	.43**	.58**	.79**	.35**	.35**	.37**	.32**	.40**	.42**	.44**
17																	1	.33**	.43**	.44**	.72**	.24**	.24**	.14*	.26**	.23**	.23**	.28**
18																		1	.29**	.41**	.64**	.16**	.12*	.14*	.19**	.18**	.16**	.19**
19																			1	.42**	.69**	0.07	0.07	.13*	0.09	0	0.07	0.09
20																				1	.78**	.21**	.18**	.22**	.23**	.27**	.12*	.24**
21																					1	.27**	.26**	.28**	.36**	.31**	.29**	.35**
22																						1	.79**	.66**	.55**	.74**	.69**	.88**
23																							1	.61**	.56**	.70**	.72**	.89**
24																								1	.51**	.62**	.63**	.79**
25																									1	.55**	.50**	.75**
26																										1	.69**	.85**
27																											1	.85**
28																												1

**Significance at p<0.01

*Significance at p<0.05.

1:ICAR-IARI 2012-13-Fe; 2:ICAR-IARI 2013-14-Fe; 3:GBPUA&T 2012-13-Fe; 4:GBPUA&T 2013-14-Fe; 5:Pusa Bihar 2012-13-Fe; 6:Pusa Bihar 2013-14-Fe; 7:Pooled-Fe; 8:ICAR-IARI 2012-13-Zn; 9:ICAR-IARI 2013-14-Zn; 10:GBPUA&T 2012-13-Zn; 11:GBPUA&T 2013-14-Zn; 12:Pusa Bihar 2012-13-Zn; 13:Pusa Bihar 2013-14-Zn; 14:Pooled-Zn; 15:ICAR-IARI 2012-13-PC; 16:ICAR-IARI 2013-14- PC; 17:GBPUA&T 2012-13- PC; 18:GBPUA&T 2013-14- PC; 19:Pusa Bihar 2012-13- PC; 20:Pusa Bihar 2013-14- PC; 21:Pooled- PC; 22:ICAR-IARI 2012-13-TKW; 23:ICAR-IARI 2013-14- TKW; 24:GBPUA&T 2012-13- TKW; 25:GBPUA&T 2013-14- TKW; 26:Pusa Bihar 2012-13- TKW; 27:Pusa Bihar 2013-14- TKW; 28:Pooled- TKW.

Fe:Grain iron concentration; Zn:Grain zinc concentration; PC:Grain protein content; TKW:Thousand kernel weight.

### Molecular mapping

**Fig 4:**
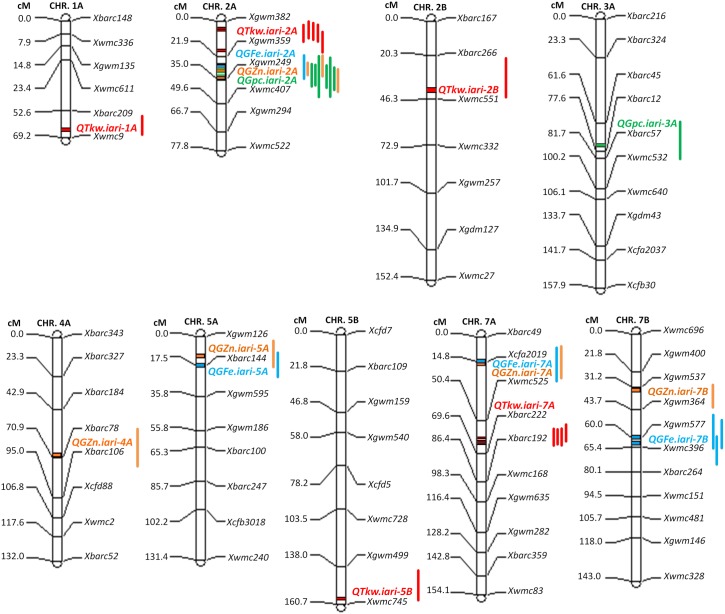
Linkage groups of wheat showing QTLs identified for grain iron, zinc, protein and thousand kernel weight by CIM in 286 RILs of the cross WH542 × Synthetic derivative. QTLs were designated using Q as abbreviation for QTL, and abbreviation of the trait name in upper case (GFe, GZn, Gpc, QTkw; Grain iron concentration, Grain zinc concentration, Grain protein content, Thousand kernel weight respectively) with a full stop, followed by Institute name (iari) in lower case and chromosome number on which the QTL is found to be located. The QTL peaks have been shown on the linkage groups and the confidence intervals by the bars (blue color for GFe, orange color for GZn, green color for Gpc and red color for GTkw).

A linkage map was constructed using 136 polymorphic SSR data of RILs. A length of 2155.3cM was covered across the 21 chromosomes. Phenotypic data obtained from all the six environments for Fe, Zn, PC, and TKW were analyzed together with genotypic data for all mapped loci assigned to the genetic maps using QTL Cartographer, version 2.1. For each of the QTLs, the closest/nearest marker was considered as the one on the linkage map closest to the peak of the QTL. A total of 16 QTLs located on chromosomes 1A, 2A, 2B, 3A, 4A, 5A, 5B, 7A, and 7B were identified in different environments (Fig 4 and Table 3). Four QTLs were identified for Fe with LOD scores ranging between 2.5 and 4.1, explaining 2.3–6.8% phenotypic variation. The four QTLs together explained 20.0% phenotypic variation for Fe, when the largest contribution of a QTL in any environment was considered. The identified QTLs for Fe were designated as *QGFe*.*iari-2A*, *QGFe*.*iari-7B*, *QGFe*.*iari-7A* and *QGFe*.*iari-5A*. *QGFe*.*iari-2A* was mapped in Pusa Bihar 2012–13 environment and the nearest marker was *Xgwm359*. *QGFe*.*iari-7B*, which was identified in two environments *viz*., Pusa Bihar 2012–13 and 2013–14, as well as in pooled mean and the nearest linked marker was *Xgwm577*. *QGFe*.*iari-5A* was mapped in pooled mean of Fe, the nearest marker identified was *Xbarc144*. *QGFe*.*iari-7A* was mapped in Pusa Bihar 2013–14 environment and the closely linked marker and flanking markers, respectively, were *Xcfa2019* and *Xbarc49-Xwmc525*.

**Table 3:** QTLs identified for grain iron, zinc, protein and thousand kernel weight in RILs of WH 542 × Synthetic derivative in rabi 2012–13 and 2013–14 at ICAR-IARI, GBPUA&T and Pusa Bihar.

Trait	Environment	QTL name	Confidence interval	Peak (cM)	Flanking markers	Nearest marker	Peak LOD	R^2^ (%)	Favorable allele^a^
**Grain Fe**	Pusa Bihar 2012–13	*QGFe*.*iari-2A*	21.9–34.9	26.9	*Xgwm359-Xgwm249*	*Xgwm359*	4.1	6.8	SD
	Pusa Bihar 2012–13	*QGFe*.*iari-7B*	44.0–65.4	60.0	*Xgwm364-Xgwm396*	*Xgwm577*	2.5	3.5	SD
	Pusa Bihar 2013–14	*QGFe*.*iari-7B*	60.2–74.5	63.6	*Xgwm577-Xbarc264*	*Xgwm577*	3.4	6.0	SD
	Pooled mean of Fe	*QGFe*.*iari-7B*	49.5–65.3	60.0	*Xgwm364-Xwmc396*	*Xgwm577*	2.8	2.5	SD
	Pusa Bihar 2013–14	*QGFe*.*iari-7A*	7.8–29.4	16.8	*Xbarc49-Xwmc525*	*Xcfa2019*	2.9	4.3	SD
	Pooled mean of Fe	*QGFe*.*iari-5A*	11.3–25.2	17.5	*Xgwm126-Xgwm595*	*Xbarc144*	3.0	2.3	SD
**Grain Zn**	ICAR-IARI 2012–13	*QGZn*.*iari-2A*	30.0–38.5	35.0	*Xgwm359-Xwmc407*	*Xgwm249*	13.5	11.1	SD
	ICAR-IARI 2013–14	*QGZn*.*iari-2A*	22.0–34.7	30.9	*Xgwm359-Xgwm249*	*Xgwm249*	11.8	14.4	SD
	Pooled mean of Zn	*QGZn*.*iari-2A*	23.6–32.9	27.9	*Xgwm359-Xgwm249*	*Xgwm359*	6.5	8.5	SD
	Pusa Bihar 2012–13	*QGZn*.*iari-4A*	58.0–76.5	69.9	*Xbarc184-Xbarc106*	*Xbarc78*	2.6	4.7	WH
	GBPUA&T 2012–13	*QGZn*.*iari-5A*	5.8–17.5	12.0	*Xgwm126-Xbarc144*	*Xbarc144*	3.7	6.2	SD
	GBPUA&T 2012–13	*QGZn*.*iari-7A*	7.0–25.2	14.8	*Xbarc49-Xwmc525*	*Xcfa2019*	2.6	3.2	SD
	ICAR-IARI 2012–13	*QGZn*.*iari-7B*	31.4–42.9	33.2	*Xgwm537-Xgwm364*	*Xgwm537*	2.4	3.4	SD
**Grain Protein Content**	ICAR-IARI 2012–13	*QGpc*.*iari-2A*	29.6–37.2	33.9	*Xgwm359-Xwmc407*	*Xgwm249*	14.6	18.8	SD
	ICAR-IARI 2013–14	*QGpc*.*iari-2A*	24.4–39.3	29.9	*Xgwm359-Xwmc407*	*Xgwm249*	5.8	9.2	SD
	GBPUA&T 2012–13	*QGpc*.*iari-2A*	25.3–38.1	29.9	*Xgwm359-Xwmc407*	*Xgwm249*	7.0	12.1	SD
	GBPUA&T 2012–13	*QGpc*.*iari-2A*	23.5–45.6	33.9	*Xgwm359-Xwmc407*	*Xgwm249*	2.9	4.3	SD
	Pusa Bihar 2012–13	*QGpc*.*iari-2A*	26.1–38.8	31.9	*Xgwm359-Xwmc407*	*Xgwm249*	5.9	9.9	SD
	Pusa Bihar 2013–14	*QGpc*.*iari-2A*	22.0–45.1	29.9	*Xgwm359-Xwmc407*	*Xgwm249*	3.0	5.4	SD
	ICAR-IARI 2012–13	*QGpc*.*iari-3A*	61.1–81.9	72.6	*Xbarc45-Xbarc57*	* Xbarc12*	2.9	5.3	SD
**Thousand kernel weight**	GBPUA&T 2012–13	*QTkw*.*iari-1A*	55.3–67.6	62.6	*Xbarc209-Xwmc9*	*Xwmc9*	3.0	6.5	SD
	ICAR-IARI 2012–13	*QTkw*.*iari-2A*	4.2–16.8	8.0	*Xgwm382-Xgwm359*	*Xgwm382*	5.9	10.4	SD
	Pusa Bihar 2012–13	*QTkw*.*iari-2A*	2.5–13.8	4.0	*Xgwm382-Xgwm359*	*Xgwm382*	3.7	6.2	SD
	Pusa Bihar 2013–14	*QTkw*.*iari-2A*	2.8–14.6	6.0	*Xgwm382-Xgwm359*	*Xgwm382*	3.3	6.0	SD
	Pooled mean of TKW	*QTkw*.*iari-2A*	3.7–15.3	8.0	*Xgwm382-Xgwm359*	*Xgwm382*	3.5	6.1	SD
	GBPUA&T 2013–14	*QTkw*.*iari-2A*	8.0–20.6	18.0	*Xgwm382-Xgwm359*	*Xgwm359*	3.9	6.4	SD
	GBPUA&T 2013–14	*QTkw*.*iari-2B*	23.7–44.8	40.3	*Xbarc266-Xwmc551*	*Xwmc551*	2.9	5.5	SD
	Pusa Bihar 2012–13	*QTkw*.*iari-5B*	140.0–158.7	156	*Xgwm499-Xwmc746*	*Xwmc746*	3.1	4.6	WH
	GBPUA&T 2012–13	*QTkw*.*iari-7A*	55.0–67.2	64.4	*Xwmc525-Xbarc222*	*Xbarc222*	3.1	5.0	SD
	Pusa Bihar 2012–13	*QTkw*.*iari-7A*	56.2–66.3	66.4	*Xwmc525-Xbarc222*	*Xbarc222*	3.4	5.3	SD
	Pusa Bihar 2013–14	*QTkw*.*iari-7A*	55.3–67.5	66.4	*Xwmc525-Xbarc222*	*Xbarc222*	3.2	4.8	SD
	Pooled mean of TKW	*QTkw*.*iari-7A*	51.6–63.4	60.4	*Xwmc525-Xbarc222*	*Xbarc222*	4.5	7.1	SD

R^2^:Phenotypic variation associated with the QTL in percentage

^a^ Favorable parental allele contributing to greater grain iron, zinc, protein and thousand kernel weight; WH:WH542 and SD:Synthetic derivative.

Five QTLs were identified for Zn in this study and these were designated as *QGZn*.*iari-2A*, *QGZn*.*iari-4A*, *QGZn*.*iari-5A*, *QGZn*.*iari-7A* and *QGZn*.*iari-7B*. The QTLs explained 3.2–14.4% phenotypic variation with LOD scores ranging between 2.4 and 13.5 and QTLs together explained 32.0% phenotypic variation for Zn. *Xgwm249* was the nearest marker linked to *QGZn*.*iari-2A* in two environments *viz*., ICAR-IARI 2012–13 and 2013–14. *QGZn*.*iari-2A* was also mapped for pooled mean and the marker nearest to the QTL was *Xgwm359*. *QGZn*.*iari-4A* was identified only in Pusa Bihar 2012–13 environment and the nearest marker was *Xbarc78*. In the GBPUA&T 2012–13 environment, two QTLs were identified (*QGZn*.*iari-5A* and *QGZn*.*iari-7A*) and the closely linked markers were, respectively, *Xbarc144* and *Xcfa2019*. *Xgwm537* was the closely linked marker to *QGZn*.*iari-7B* in ICAR-IARI 2012–13 environment. Two QTLs (*QGpc*.*iari-2A* and *QGpc*.*iari-3A*) were identified for PC in this study. *QGpc*.*iari-2A* was mapped in all the six environments and the nearest linked marker and flanking markers, respectively, were *Xgwm249* and *Xgwm359-Xwmc407*. The other QTL identified for PC was mapped between flanking markers of *Xbarc45* and *Xbarc57* on chromosome 3A. The QTLs explained between 4.3–18.8% phenotypic variation with LOD score ranging between 2.9–14.6 in different environments. The two QTLs (*QGpc*.*iari-2A* and *QGpc*.*iari-3A*) together explained 24.1% phenotypic variation. The markers *Xgwm359*, *Xbarc144* and *Xcfa2019* were associated with both Fe and Zn, whereas the marker *Xgwm249* was linked to both Zn and PC. A genomic region in the interval of *Xgwm359-Xwmc407* was associated with Fe, Zn, and PC.

Five QTLs were identified for TKW and were designated as *QTkw*.*iari-1A*, *QTkw*.*iari-2A*, *QTkw*.*iari-2B*, *QTkw*.*iari-5B* and *QTkw*.*iari-7A*. The QTLs explained 4.6–10.4% phenotypic variation with LOD scores ranging between 2.9 and 5.9 and QTLs together explained 32.3% phenotypic variation for TKW. *QTkw*.*iari-2A* was identified in four environments *viz*., ICAR-IARI 2012–13, GBPUA&T 2013–14, Pusa Bihar 2012–13 and 13–14. *QTkw*.*iari-2A* was also mapped for pooled mean and the marker nearest to the QTL in all the environments except in GBPUA&T 2013–14 was *Xgwm382*. *QTkw*.*iari-7A* was identified in three environments *viz*., GBPUA&T 2012–13, Pusa Bihar 2012–13 and 2013–14 apart from pooled mean. The nearest linked marker and flanking markers, respectively, were *Xbarc222* and *Xwmc525-Xbarc222*.

*QTkw*.*iari-1A* was mapped in GBPUA&T 2012–13 environment and the closely linked marker and flanking markers, respectively, were *Xwmc9* and *Xbarc209-Xwmc9*. *Xwmc551* was the nearest linked marker for *QTkw*.*iari-2B* that was identified in GBPUA&T 2013–14 environment within the marker interval of *Xbarc266-Xwmc551*. In the Pusa Bihar 2012–13 environment, one QTL was identified (*QTkw*.*iari-5B*) and the closely linked marker and marker interval, respectively, were *Xwmc746* and *Xgwm499-Xwmc746*. A low TKW can result from shriveled grains where the bran fraction may be higher. Since bran is richer in micronutrients as compared to the starchy endosperm, it is important to know if the parent that contributed favorable alleles for high for grain micronutrients was also the parent for contributing favorable alleles for TKW.

All the QTLs identified in the present study had favorable alleles (allele that increases Fe, Zn, PC and TKW) contributed by the high parent with the exception of *QGZn*.*iari-4A* and *QTkw*.*iari-5B*, where favorable allele was contributed by the low parent WH542 (Table 3).

## Discussion

RILs showed a wide variability for Fe, Zn, PC, and TKW in different environments. The relative contribution of genotype, environment and genotype-environment interaction on the Fe and Zn traits of this RIL population has been studied [18]. The positive and highly significant associations among Fe, Zn, PC and TKW found in this study have been reported in different wheat populations in earlier studies [33, 53–54]. The distribution of values of Fe, Zn, PC, and TKW in different frequency classes implied that these traits were controlled by polygenes. Therefore, QTL mapping was carried out. Alleles responsible for increased Fe at all the QTLs were inherited from the high parent. Four QTLs (*QGFe*.*iari-2A*, *QGFe*.*iari-5A*, *QGFe*.*iari-7A* and *QGFe*.*iari-7B*) were identified for increased Fe. In earlier studies also, QTLs were identified for Fe on chromosome 2A [30–34], 5A [32–33], 7A [33–34] and 7B [31, 33] in RIL populations. In this study, an additional QTL (*QGFe*.*iari-7B)* was expressed in both the environments of Pusa Bihar 2012–13 and 2013–14, and also for pooled mean. Five QTLs for Zn (*QGZn*.*iari-2A*, *QGZn*.*iari-4A*, *QGZn*.*iari-5A*, *QGZn*.*iari-7A and QGZn*.*iari-7B*) were identified in the present study. Earlier reports for Zn on chromosome 2A [30, 32–33], 4A [31, 37], 5A [32–33, 37], 7A [33–35, 37] and 7B [33] in different mapping populations corroborate the involvement of these chromosomes. Two QTLs (*QGpc*.*iari-2A* and *QGpc*.*iari-3A*) were identified for PC. Earlier studies also reported QTLs for PC [27, 38–39, 55–60] on several different chromosomes. Five QTLs (*QTkw*.*iari-1A*, *QTkw*.*iari-2A*, *QTkw*.*iari-2B*, *QTkw*.*iari-5B* and *QGFe*.*iari-7A*) were identified in our study for TKW. Earlier reports for TKW on chromosome 1A [40, 44], 2A [41], 2B [40, 44], 5B [40, 44] and 7A [41, 43, 45] in different mapping populations confirms the involvement of these chromosomes.

A common region between *Xgwm359-Xwmc407* on chromosome 2A was associated with the increase of Fe, Zn and PC and the identified QTLs were co-located. Some of the above-mentioned studies have also identified the common region(s) associated with both Fe and Zn and among Fe, Zn and PC [26–27]. QTLs for all the studied traits (Fe, Zn, Protein, and TKW) on chromosome 2A and for three traits (Fe, Zn, and TKW) on chromosome 7A were identified. Highest phenotypic variation associated with the QTL as expressed by R^2^ was also reported in 2A. Therefore, common regions on chromosome 2A and 7A harbours the QTLs for all the studied traits and could be prominent candidate chromosome regions for traits improvement. Though some of the chromosomes and the genomes are common among different studies, the latter differ with respect to the genomic regions harbouring the QTLs. Apart from soil variations in terms of nutrient distribution and their availability in the soil prompting the plants to use different mechanisms for the uptake of nutrients, the level of contamination that may result in sampling and actual experimentation may also cause shifts in the chromosomal locations of the QTLs [61].

While twelve QTLs for the four traits were mapped on A genome, only four were detected on B genome and none on the D genome in this study. The same order of the frequency of involvement of genomes was observed in earlier studies also.

## Conclusion

The study with 286 RILs developed by single seed descent from the cross between a cultivated bread wheat variety and a synthetic derivative has shown that Fe, Zn, PC, and TKW are quantitatively inherited traits and their expression is greatly influenced by environment. The strong positive association among Fe, Zn, PC, and TKW suggest that improving one of the traits would allow the others to be improved simultaneously. Four, five, two and five QTLs were identified, respectively, for Fe, Zn, PC, and TKW. The QTLs identified in the present investigation represent novel genomic regions associated with Fe, Zn, PC, and TKW. The presence of a common locus on chromosome 2A associated with Fe, Zn and PC, and one QTL identified at different chromosomal region for TKW on the same 2A chromosome, as well as of the common loci on chromosome 5A and 7A associated with both Fe and Zn, along with one QTL identified at different chromosomal region for TKW on the same 7A chromosome imply that at least three chromosomes of A genome are important in determining accumulation of Fe, Zn, PC and TKW and, therefore, fine mapping of the involved regions in these chromosomes may be rewarding.

## Supporting information

S1 Table Individual mean values of RILs for Fe tested in six environments. (XLSX)

S2 Table Individual mean values of RILs for Zn tested in six environments. (XLSX)

S3 Table Individual mean values of RILs for PC tested in six environments. (XLSX)

S4 Table Individual mean values of RILs for TKW tested in six environments. (XLSX)
